# Analysis of the Function of Apoptosis during Imaginal Wing Disc Regeneration in *Drosophila melanogaster*

**DOI:** 10.1371/journal.pone.0165554

**Published:** 2016-11-28

**Authors:** Sandra Diaz-Garcia, Sara Ahmed, Antonio Baonza

**Affiliations:** 1 Centro de Biología Molecular “Severo Ochoa”, CSIC and Universidad Autónoma de Madrid, Madrid, Spain; 2 University of California San Diego, Biology Section of Cell & Developmental Biology, United States of America; University of Dayton, UNITED STATES

## Abstract

Regeneration is the ability that allows organisms to replace missing organs or lost tissue after injuries. This ability requires the coordinated activity of different cellular processes, including programmed cell death. Apoptosis plays a key role as a source of signals necessary for regeneration in different organisms. The imaginal discs of *Drosophila melanogaster* provide a particularly well-characterised model system for studying the cellular and molecular mechanisms underlying regeneration. Although it has been shown that signals produced by apoptotic cells are needed for homeostasis and regeneration of some tissues of this organism, such as the adult midgut, the contribution of apoptosis to disc regeneration remains unclear. Using a new method for studying disc regeneration in physiological conditions, we have defined the pattern of cell death in regenerating discs. Our data indicate that during disc regeneration, cell death increases first at the wound edge, but as regeneration progresses dead cells can be observed in regions far away from the site of damage. This result indicates that apoptotic signals initiated in the wound spread throughout the disc. We also present results which suggest that the partial inhibition of apoptosis does not have a major effect on disc regeneration. Finally, our results suggest that during disc regeneration distinct apoptotic signals might be acting simultaneously.

## Introduction

Regeneration allows organisms to restore the original shape, size and function of body parts that have been lost or damaged. The imaginal wing discs of *Drosophila melanogaster* have the capacity to regenerate during the larval stages and provide a particularly well-characterised model system for analysing this phenomenon (review [1]). The imaginal wing discs are sac-like structures that give rise to the wing and notum of the adult. The cells that constitute the discs are specified early in embryogenesis. They start to divide during the first larval stage and continue proliferating until the end of larval development. Since a series of classic experiments by Ernst Hadorn (1940s to the 1970) [2–4] laid the basis for understanding imaginal disc regeneration, different experimental approaches have been used to study this process in *Drosophila*. Classically disc regeneration has been studied in amputated discs that were cultivated into the abdomen of an adult host where the cells of the discs proliferate but do not differentiate [2,5,6]. More recently, a new system based on the *Gal4/UAS* binary system, in combination with a temperature-sensitive Gal4 suppressor, Gal80^ts^, has been developed to genetically ablate a region of the wing disc. This method allows the induction of cell death in specific domains of the discs for a limited period of time, after which the discs recover [7–8]. The results obtained from these studies have provided fundamental principles for a variety of cellular and molecular processes involved in organ regeneration, such as tissue remodelling, migration, cell de-differentiation, patterning, and control of cell proliferation (review in [1, 9,10]). All these processes must be precisely regulated and coordinated during regeneration to restore the size and pattern of the damaged organ. Recently different reports have shown that apoptosis plays essential functions during animal regeneration [11–13]. One of the model organisms that has provided the most compelling evidence for the contribution of apoptosis in regeneration is the freshwater polyp Hydra [14]. When the body of the Hydra is transversally sectioned, apoptosis is triggered only in the lower half, that is the fragment that will re-grow a head, whereas it is undetectable in the upper part that will form a new foot. The ectopic induction of cell death in the upper part induces head formation and gives rise to a doubled-headed Hydra [14]. Apoptosis has also been shown to function during regeneration in vertebrate animals such as in the case of Xenopus tadpoles [15] and in wound healing and liver regeneration in mice [16–17].

In Drosophila the role of cell death during disc regeneration remains largely unknown. The different experimental procedures used to study disc regeneration present different problems and have led to inconclusive results. Disc cutting and transplantation assays suggest that apoptosis does not play an important role during disc regeneration, as disc regeneration seems to proceed normally when cell death is suppressed by the over-expression of the baculoviral caspase inhibitor p35 [18]. However, these experiments do not allow the analysis of the pattern and size of the adult regenerated wings, thus it is not possible to determine whether regeneration is normally completed. Furthermore, disc transplantation and *in vivo* culture conditions increases the number of dead cells throughout the discs, even in control non-amputated discs [18–19], therefore it remains unknown whether apoptosis increases during disc regeneration.

The genetic ablation experiments rely on the expression of pro-apoptotic genes in a specific region of the discs; consequently cell death cannot be blocked in the targeted region, and it is not possible to examine the effects that this produces during regeneration [7–8]. Moreover, the ectopic expression of the pro-apoptotic genes may promote different cellular responses not associated with regeneration; for instance it has been shown that apoptotic cells can induce non-autonomous cell death in neighbouring cells [20]. Accordingly, a non-autonomous increase of cell death in regenerating discs might be caused by the ectopic induction of the apoptotic genes and not directly by the regeneration process. Thus, it still remains to be determined whether apoptosis is involved in disc regeneration [12, 20].

We recently have developed a method to study disc regeneration *in vivo* in physiological conditions [21]. With this system is possible to study disc regeneration in its normal developmental context. Furthermore, it is also possible to examine the possible effects of blocking apoptosis on determination of the final size and pattern of the adult regenerating wings. We have taken advantage of this method to define the pattern of apoptosis at different times during disc regeneration. Our results indicate that in regenerating discs, cell death increases firstly at the wound edge, but as regeneration progresses apoptosis is extended throughout the disc. We have also found that partial inhibition of apoptosis does not have a major effect on disc regeneration. Finally, our data also suggest that during disc regeneration distinct mechanisms to induce cell death might be cooperating.

## Materials and Methods

### *Drosophila* stocks and genetics

The following stocks and *Gal4* lines were used:

The UAS lines used included: *UAS-GFP* (Bloomington stock center), *UAS-hep*^*CA*^ *(II)* [22], *UAS-puc2A* [23], *UAS-Diap1(III)* [24], *UAS-p35(III)* (Bloomington stock center), *and UAS-eiger* (a gift from G. Morata) [25].

We used the Gal4 lines *enGal4 UAS-GFP/CyO*, *nubGal4/CyO*, We obtained these lines from Gines Morata (CBM, Madrid). *Hh-dsRed Ci-Gal4 UAS-GFP/TM6B* (a gift from Carlos Estella). All of these stocks have been previously described in FlyBase (http://flybase.bio.indiana.edu/).

The reporter puc-LacZ line [23].

The flies stocks *eiger*^*1*^*/Cyo and eiger*^*3*^*/S-T* (a gift from Hermann Steller) [20].

### Immunocytochemistry

Immunostaining of the wing discs was performed according to standard protocols. The following antibodies were used: rabbit anti-cleaved Caspase-3 (Cell Signaling); rabbit anti-Phospho-histone 3 (Upstate; 1:1000); mouse anti-CD2 (1:100; Serotech mouse anti- ß -Galactosidase (Promega Z3778A; 1:200); mouse anti-Dl (C594.9B; 1:50); and mouse anti-Wg (4D4; 1:100), were obtained from the Developmental Studies Hybridoma Bank at the University of Iowa. Secondary antibodies (Molecular Probes) were used at dilutions of 1:200.

Analysis of cell proliferation and the expression patterns of different markers in control animals were performed by crossing *en-Gal4 UAS-GFP/CyO* flies to wildtype (wt).

### Analysis of regeneration under conditions that prevented apoptosis

This analysis was performed by crossing *w; en-Gal 4 UAS-GFP/CyO* to *w; nub-Gal4; UAS p35 (III)* or to *w; nub-Gal4; UAS-dIAP1(III) or to UAS-puc (II)*. To study *eiger* function during regeneration *eiger*^*1*^*; Hh-dsRe Ci-Gal4 UAS-GFP/S-T* flies were crossed *to eiger*^*3*^*/ eiger*^*3*^.

Larvae were raised at 25°C before the surgical elimination of the fragment and were maintained at that temperature for the rest of the development.

### Ectopic activation of JNK signalling

This analysis was performed by crossing *w; en-Gal 4 UAS-GFP/CyO; tub Gal80*^*ts*^*/ TM6b* to *w; UAS- hep*^*CA*^ *(II)* or to *w; UAS-eiger*.

*w; en-Gal 4 UAS-GFP/UAS- hep*^*CA*^*; tub Gal80*^*ts*^*/ +* or with *w; UAS-eiger* larvae were raised at 17°C until 240 hrs after egg laying (AEL) and then shifted to 29°C for 24 hrs before analyzing the expression of Cas3* or PH3. To study ectopic activation of JNK in *eiger* mutants, *eiger*^*1*^*; Hh-dsRed Ci-Gal4 UAS-GFP/S-T* flies were crossed to *eiger*^*3*^*; pucLacZ/TM6B*.

### Imaginal disc manipulation and analysis

Surgical ablation was performed on mid-late third instar *en-Gal4 UAS-GFP* larvae or *w; en-Gal 4 UAS-GFP/+; nub-Gal4/+* (120–140 hrs AEL). Unless otherwise indicated larvae were raised at 25°C. The expression of *UAS-GFP* in the posterior compartment or the entire wing pouch driven by *en-Gal4* or *nub-Gal4* respectively enabled the identification of the discs inside the larvae using a binocular microscope (Leica MZFLIII) and UV light. A section of the posterior or anterior/posterior compartment was removed by closing a pair of forceps over the wing disc without breaking the larval cuticle. The larvae were maintained on ice during this process.

### In vitro culture

Imaginal discs were cultured as described [26] except that we do not add Ecdysone to the medium. The discs cultivated were *eiger*^*1*^ */ eiger*^*3*^*; pucLacZ/+ or controls pucLacZ*.

### Mitotic and apoptotic index

We calculated the mitotic and apoptotic index as the average value of the ratio between the number of cells in mitosis or dying in the posterior or anterior compartments, as detected by the expression of phospho-histone3 (PH3) and Caspase-3, respectively, and the size of the compartment in μm (PH3-positive or Cas3* cells/size of the compartment). We analyzed at least 5 discs for each experiment. We only considered the wing blade and hinge territories.

## Results

### Pattern of cell death during wing disc regeneration

**Fig 1:**
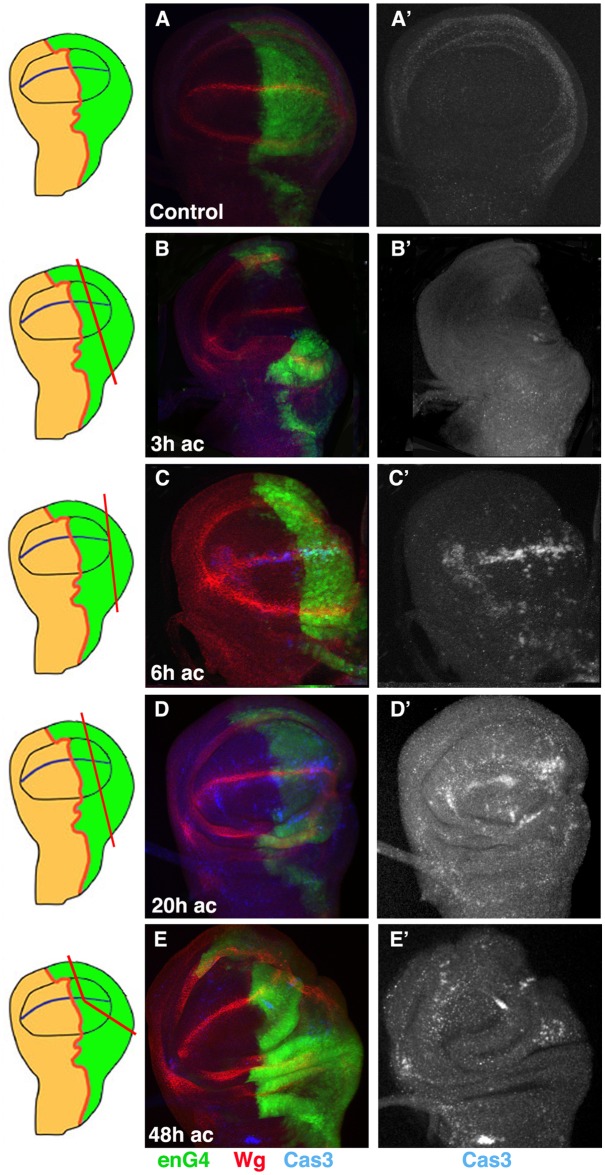
Pattern of cell death during wing disc regeneration. (A-E’) Third instar wing *en-Gal4 UAS-GFP* discs stained for the apoptotic marker anti-cleaved Caspase-3 (blue in A-E, and grey in A’-E’) and anti-Wg (red A-E). (A-A’) Control discs. (B-B’) Regenerating discs at 3 hrs after cut (AC). We only observed a few dead cells at the wound edge or in the region adjacent. (C-C’) Regenerating discs at 6 hrs AC; we observed a significant increase in the number of dead cells in the posterior, as well as the anterior compartments. Note the increased number of dying cells along the d/v boundary. (D-D’) 20 hrs AC, we still observed a high number of apoptotic cells throughout the wing disc. (E-E’) 48 hrs AC, there is a reduction in the number of dead cells in both compartments. Schematic illustrations on the left indicate the cutting lines and the regions eliminated in each disc.

**Fig 2:**
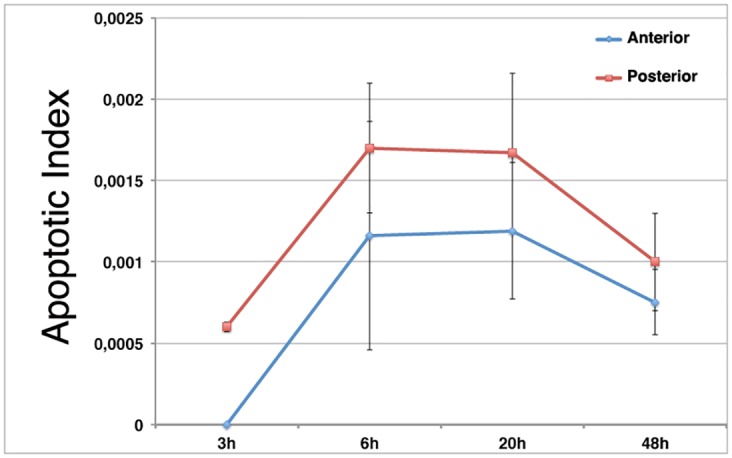
Cell death during wing disc regeneration. Bar chart shows the average apoptotic index in the posterior (black square) and anterior (grey) compartments of regenerating discs at different times AC. The error bars represent the standard deviation.

The contribution of cell death to disc regeneration has remained elusive, as none of the experimental approaches used to study this process have allowed it to be defined [18]. We first have examined the parameters of cell death in regenerating discs using a method developed by us that allows the study of disc regeneration in its normal developmental context [21]. Using this system we have amputated a fragment of the posterior compartment of *en-Gal4 UAS-GFP* wing discs, and analysed discs labelled with cleaved Caspase-3 (Cas3*) antibody at different times after amputation; as a control we used the contra-lateral non-amputated wing discs. Surgical sections were inflicted in third instar larval discs (120–140 hrs AEL). We observed that during the first 3 hrs of regeneration the number of dead cells was not significantly increased in the wing blade region, and we only found a few dead cells close to the wound edge (Figs 1 and 2). However, at 6 hrs after amputation (AC), the number of dead cells strongly increased at the wound edge as well as in regions adjacent to it, extending along the DV (Figs 1 and 2, and S1 Fig). Interestingly, even though we only eliminated a fragment of the posterior compartment, we observed that Caspase-3 is also expressed in the anterior compartment, in regions far away from the wound edge (Fig 1, S1 and S2 Figs). To confirm that the Caspase-3 staining in the anterior compartment corresponds to apoptotic cells and not to cellular debris or to posterior apoptotic cells that have moved away from the wound site, we double stained regenerating discs for Capase-3 and DAPI. As seen in transverse sections (S2 Fig), we found Caspase-3 positive cells in the anterior compartment that are still integrated in the columnar epithelium. In addition, in contrast to posterior dying cells, that always expressed GFP, the anterior apoptotic cells do not express this marker. All these data indicate that during regeneration cell death can be induced in regions far away from the wound.

We found similar results in wing discs analysed at later stages (20 hrs AC) (Figs 1 and 2, S1 and S2 Figs). At this stage we frequently observed clusters of dead cells in the anterior compartment (Fig 1 and S2 Fig). The increasing number of dead cells decreased in discs analysed at 48 hrs AC (Figs 1 and 2). The elimination of a fragment of the anterior compartment causes similar defects to those observed when we amputate part of the posterior compartment [21].

Altogether, our data indicate that during disc regeneration, apoptosis is first activated at the wound edge and in adjacent regions, but as regeneration progresses an apoptotic signal, or mechanical tension, induces cell death throughout the wing disc.

### Ectopic expression of DIAP is not sufficient to block cell death during regeneration

**Fig 3:**
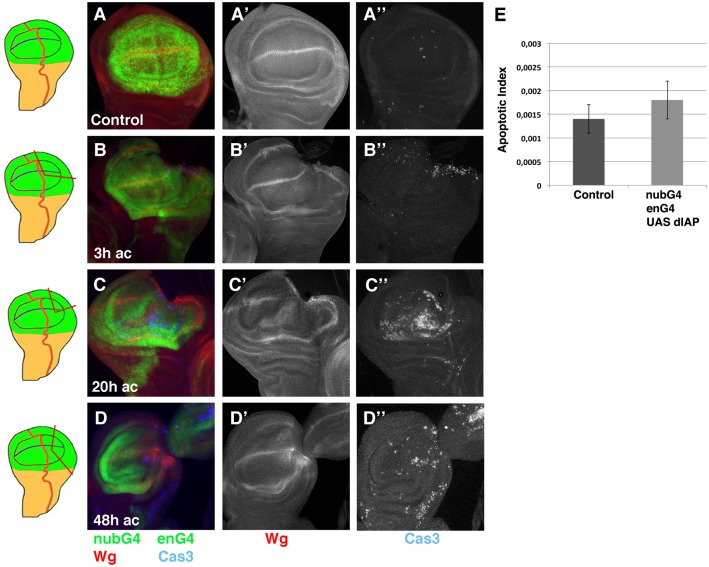
The over-expression of *dIAP* is not sufficient to block apoptosis during disc regeneration. (A-D”) Third instar wing *en-Gal4 UAS-GFP* / *nub-Gal4; UAS-dIAP1*/+ discs stained for anti-cleaved Caspase-3 (blue in A-D, and grey in A”-D”) and anti-Wg (red A-D and grey A’-D’). (A-A”) Control *en-Gal4 UAS-GFP*/*nub-Gal4; UAS-dIAP1*/+ disc. (B-B”) Regenerating *en-Gal4 UAS-GFP* / *nub-Gal4; UAS-dIAP1*/+ discs at 3 hrs AC. As in control discs, we found a few dead cells at the wound edge or in the region adjacent. (C-C”) 20 hrs AC, we observed a high number of dead cells throughout the wing blade. (D-D”) 48 hrs AC, as observed in control discs, at this time there is a reduction in the number of dead cells in both compartments. (E) Bar chart shows the average apoptotic index in control regenerating discs (Control) and *en-Gal4 UAS-GFP* / *nub-Gal4; UAS-dIAP1*/+ (*nubG4 enG4 UAS dIAP1*) discs at 20hrs AC. Here and in the rest of figures the error bars represent the standard deviation. Schematic illustrations on the left indicate the cutting lines and the regions eliminated in each disc.

We explored the possible requirement of apoptosis during disc regeneration by analysing the effects caused by the inhibition of cell death in regenerating discs. To this end we over-expressed different factors that suppressed cell death. One important family of Caspase inhibitors are the IAP (Inhibitor of Apoptosis Proteins), which can bind to and inhibit Caspases. In cells that are committed to die, the proapoptotic genes Reaper, Hid, and Grim inhibit the activity of the *Drosophila* IAP1 (dIAP). We first tried to block cell death in regenerating discs by over-expressing *dIAP*. As described above, in regenerating control discs cell death increases not only in the region near to the wound edge, but also in regions far away from it. Thus, we observed apoptotic cells in the anterior compartment, even though we only amputated a fragment of the posterior compartment. Therefore, to better evaluate the role of cell death during regeneration we over-expressed dIAP1 in the entire wing pouch. To this end we expressed *UAS-dIAP1* under the control of *en-Gal4* together with nub-Gal4, which drives the expression of Gal4 in the wing pouch. We amputated part of the posterior or anterior compartment of these discs and analysed the spatial and temporal pattern of cell death and proliferation in regenerating wing discs at different times AC. We found that at the different times analysed, the distribution of Caspase-3 positive cells in amputated discs that over-express dIAP1 is very similar to that observed in control amputated discs (Fig 3 compared with Fig 1) (Apoptotic index in the wing blade of these discs at 20 hrs AC is 0,0018±0,0004, n = 5 *vs* 0,0014±0,0003 in Control regenerating discs n = 8). As previously reported for control amputated discs [21], we observed that between 18–24 hours after amputation, cell proliferation increases in the region near to the wound edge, as assayed with the mitotic marker (PH3) (Mitotic index 0,0037±0,0007 in regenerating discs that over-expressed dIAP1, n = 7 *vs* 0,0021 ± 0,0005 in control non amputated contralateral discs, n = 7, p<0.01). The increased proliferation in these discs is similar to that observed in control regenerating discs (1,76 ±0,3 vs 1,7± 0,1 in control regenerating discs) (S3 Fig) [21]. Moreover, as it occurs in control regenerating wings discs between 18–20 hrs AC, the expression of Wingless (Wg) disappears from part of the dorsal-ventral (d/v) boundary in 69% (n = 9) of the wing discs analysed (in control regenerating discs Wg disappear in 76% n = 29). The size of adult regenerated wings derived from these discs is comparable to control regenerated wings (data not shown). All these data imply that the ectopic expression of dIAP1 is not disturbing disc regeneration, in addition they also suggest that the over-expression of dIAP1 is not sufficient to block apoptosis during disc regeneration.

### JNK signalling is required to induce apoptosis during disc regeneration

Our results indicate that regeneration promotes cell death not only in the region adjacent to the wound edge, but also in regions that were away from it. This effect suggests the existence of one or more signals that are activated near the damaged epithelia and that spread throughout the rest of the disc. Different reports have shown that the Jun-N terminal Kinase (JNK) signalling pathway plays an important role during regeneration and its function is also involved in the induction of apoptosis [19, 27–31]. During disc regeneration, this pathway is initially activated in the cells that form the wound edge, but as regeneration progresses its activity expands to most of the cells within the disc [19] (S4 and S5 Figs). Recently, it has been shown that apoptotic cells can also induce non-autonomous cell death in neighbouring cells and tissues [20]. Interestingly this process, that has been termed apoptosis-induced apoptosis, relies on the production of the TNF ortholog Eiger by apoptotic cells [20]. This factor activates the JNK pathway in neighbouring cells and in adjacent regions, inducing them to die [12,20]. All these results are consistent with a possible function of this signaling pathway in the induction of apoptosis in cells adjacent to the wound as well as in regions far away from it during regeneration.

**Fig 4:**
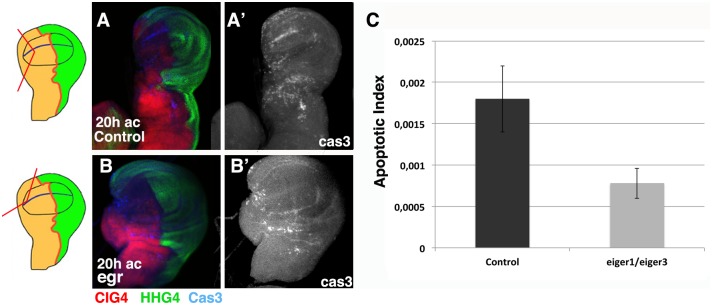
Apoptosis is reduced in *egr*^*1*^*/egr*^*3*^ regenerating discs. (A-B’) Third instar wing discs stained for the apoptotic marker anti-cleaved Caspase-3 (blue in A-B, and grey in A’-B’). (A-A”) Control *HhdsRed-CiGal4 UAS-GFP/+* regenerating discs amputated in the anterior compartment. (B-B”) Regenerating *eiger*^*3*^*/eiger*^*1*^; *HhdsRed Ci-Gal4 UAS-GFP/+* discs at 20 hrs after cut. We found that there is a reduction in the number of dead cells in both compartments. (C) Bar chart shows the average apoptotic index in control regenerating discs amputated in the anterior compartment (Control) and *eiger*^*3*^*/eiger*^*1*^; *HhdsRed Ci-Gal4 UAS-GFP/+* (*eiger*^*3*^*/eiger*^*1*^) regenerating discs at 20 hrs AC. Note that the apoptotic index of discs amputated in the anterior compartment is very similar to that observed in discs amputated in the posterior compartment (see Fig 2). Schematic illustrations on the left indicate the cutting lines and the regions eliminated in each disc.

**Fig 5:**
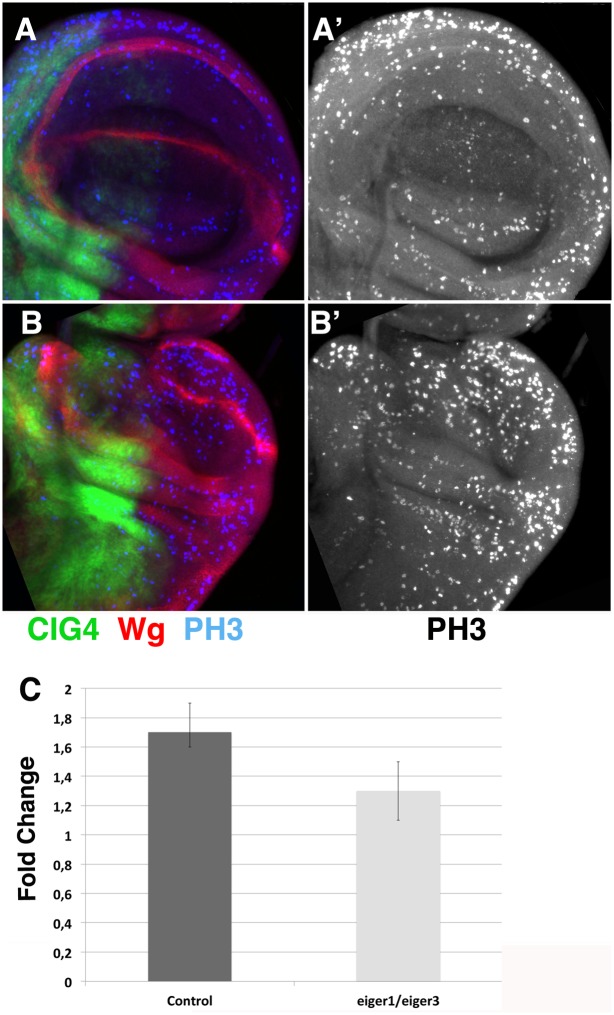
Regenerative growth is reduced in *egr*^*1*^*/egr*^*3*^ regenerating discs. (A-B’) Third instar wing discs stained for the mitotic marker phospho-Histone H3 (blue in A-B, and grey in A’-B’) and anti-Wg (red in A-B). (A-A’) Control *HhdsRed Ci-Gal4 UAS-GFP/+* disc. (B-B’) Regenerating *eiger*^*3*^*/eiger*^*1*^; *HhdsRed Ci-Gal4 UAS-GFP/+* discs at 20 hrs after cut. We found that there is a reduction in the number of mitotic cells in both compartments. (C) Bar charts show the average fold change in the mitotic index of control regenerating discs (control), and *eiger*^*3*^*/eiger*^*1*^;*HhdsRedCi-Gal4 UAS-GFP/+* regenerating discs (*eiger*^*3*^*/eiger*^*1*^) at 20 hrs AC, compared to control non-regenerating discs.

We explored the possible function of the JNK signalling pathway promoting cell death during regeneration by analysing the expression of Caspase-3 in regenerating *eiger* loss of function mutant discs. To this end we amputated a fragment of *eiger*^*3*^ */eiger*^*1*^; *Hh-dsRed Ci-Gal4 UAS-GFP/+* wing discs and analysed the apoptotic pattern. We found that in these discs cell death was strongly reduced at 20 hrs AC compared to control regenerating discs (Apoptotic index is 0,0018±0,0004, n = 5 in control regenerating discs *vs* 0,00077±0,00018, n = 5 in *eiger*^*3*^ */eiger*^*1*^; *Hh-dsRed Ci-Gal4 UAS-GFP/+* regenerating discs, p<0.01) (Fig 4). However, we still observed a significant number of dead cells specifically in the region close to the wound site (Fig 4 and S6 Fig). We also have examined the pattern of cell proliferation in *eiger*^*3*^*/eiger*^*1*^ mutant regenerating discs. We found that in contrast to control regenerating discs, that at 20 hrs AC show a strong increase of cell proliferation in the damaged region (1,7±0,1, n = 7 higher than in control non-damaged region), in *eiger*^*3*^*/eiger*^*1*^ mutant regenerating discs cell proliferation is only slightly increased (1,3 ± 0,2 n = 4, p<0.01) (Fig 5). In addition, we found that 6 hrs AC the amputated *eiger*^*3*^*/eiger*^*1*^ discs are unhealed, at this time wound healing is completed in control discs (S4 Fig). Finally in contrast to control regenerating discs, that show a partial loss of Wg expression at 20 hrs AC (see above) [21], in *eiger*^*3*^*/eiger*^*1*^ regenerating discs the expression of Wg remains unaffected during regeneration (S6 Fig). All these data suggests that in the *eiger*^*3*^*/eiger*^*1*^ mutant, disc regeneration is arrested or delayed. This is consistent with previous reports which indicate that the over-expression of the phosphatase *puckered (puc)*, which controls the activity of JNK signalling by a negative feedback loop [23], inhibits or delays wound healing and regeneration [19]. We found similar results when *puc* is over-expressed in regenerating discs that were amputated using our method (S7 Fig).

Our data suggest that *eiger* plays an important role in activating autonomous and non-autonomous apoptosis during disc regeneration. However, the presence of numerous dead cells in regenerating *eiger* mutant discs, in contrast with the complete suppression of apoptosis in these mutant discs after cell death induction [20] implies that during disc regeneration, in addition to JNK signalling, other signaling pathways might be involved in the induction of apoptosis. Alternatively, JNK might be activated by other mechanisms independently of Eiger, or both processes might be operating together during disc regeneration. The JNK signalling pathway can be triggered via a number of means in different contexts [32–33], therefore we first examined the activity of JNK signalling in *eiger*^*3*^*/eiger*^*1*^ regenerating discs. To this end we used a LacZ insertion in the gene *puckered (puc)*. As previously reported, we observed that in regenerating control discs this reporter was activated firstly at the wound edge, but later expanded to most of the cells of the wing blade [19] (S4 and S5 Figs). Interestingly, we found that although the expression of *puc-LacZ* is strongly down-regulated in regenerating *eiger*^*3*^*/eiger*^*1*^ discs compared to control regenerating discs, we observed some cells at the wound edge that express high levels of this reporter (analysed at 6 hrs and 20 hrs AC) (S4 and S5 Figs). These data suggest that in addition to Eiger, other mechanisms and signals activate JNK signalling during disc regeneration at the wound edge.

**Fig 6:**
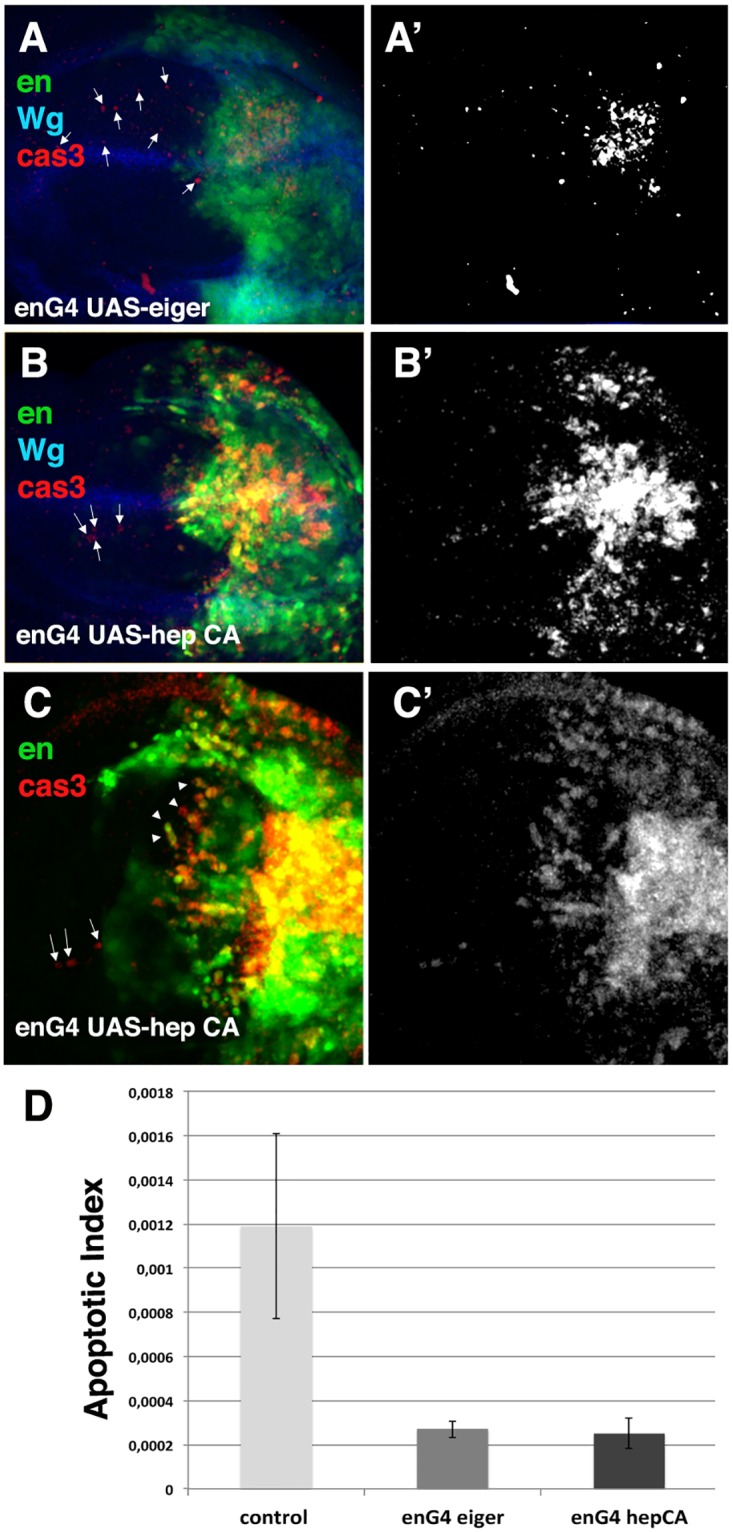
Ectopic activation of JNK signalling is not sufficient to induce the”non autonomous” cell death observed in regenerating control discs. (A-C’) Third instar *en*-*Gal4 UAS-eiger UAS-GFP /Tub-Gal80*^*ts*^ wing discs (A-A’), and *en*-*Gal4 UAS-hep*^*CA*^ *UAS-GFP /Tub-Gal80*^*ts*^ wing discs (B-C’). Larvae were shifted from 17°C to 29°C for 24 hrs before the staining. The discs were stained with anti-cleaved Caspase-3 (red in A-C, and grey in A’-C’); anti-Wg (blue A-B). (A-A’) The ectopic expression of *eiger* in the posterior compartment only induces the apoptosis of a few scattered cells in the anterior compartment (arrows), compared with regenerating discs Fig 1. (B-B’) Although cell death is massively induced in the posterior compartment of *en*-*Gal4 UAS-hep*^*CA*^ *UAS-GFP /Tub-Gal80*^*ts*^ discs, we only found a few apoptotic cells in the anterior compartment (arrows). (C-C’) High magnification of *en*-*Gal4 UAS-hep*^*CA*^ *UAS-GFP /Tub-Gal80*^*ts*^ discs. Most apoptotic cells in the A/P boundary are posterior cells, since they also express GFP (arrowheads), only a few dead cells are anterior (arrows). (D) Bar chart shows the average apoptotic index in the anterior compartment of control regenerating discs (control), *en*-*Gal4 UAS-eiger UAS-GFP /Tub-Gal80*^*ts*^ (enG4 eiger) and discs *en*-*Gal4 UAS-hep*^*CA*^ *UAS-GFP /Tub-Gal80*^*ts*^ (enG4 hepCA). Cell death index 0,000251323±6,98223E-05, n = 5 and 0,00027647 ± 3,84519E-05, n = 5 in the anterior compartment of discs over-expressing *hep*^*CA*^ and *eiger* respectively vs 0,00119 ±0,00042,n = 8 in the anterior compartment of control regenerating discs.

The existence of apoptotic cells in regenerating *eiger* mutant discs far away from the wound edges suggest that the induction of apoptosis during disc regeneration does not only depend on the apoptosis-induced apoptosis mechanism triggered by Eiger. Consistent with this hypothesis, we found that the temporary ectopic expression of *eiger* using the Gal4/Gal80^Ts^ system (see M&M), causes massive apoptosis in the targeted region (posterior compartment, autonomous cell death), but only induces the apoptosis of a few cells in the anterior compartment (non autonomous cell death) (cell death density 0,00027647 ± 3,84519E-05, n = 5 in the anterior compartment of discs over-expressing *eiger* vs 0,00119 ±0,00042,n = 8 in the anterior compartment of control regenerating discs) (Fig 6). Thus, cell death was 4 times higher in the anterior compartment of control regenerating discs than in discs over-expressing *eiger*. Moreover, in contrast to control regenerating discs, most of the dead cells in the anterior compartment of these mutant discs were adjacent to the anterior/posterior boundary (Fig 6 compare to Fig 1). We have found that the ectopic activation of JNK signalling by the over-expression of an activated form of *hemipterous (hep*^*CA*^*)* [34] cause similar effects (cell death density 0,000251323±6,98223E-05, n = 5 in the anterior compartment of discs over-expressing *hep*^*CA*^ vs 0,00119 ±0,00042,n = 8 in the anterior compartment of control regenerating discs) (Fig 6). Altogether our results imply that the activation of JNK signaling is not sufficient alone to induce the strong pattern of apoptosis in regions both adjacent and far away from the wound observed in regenerating discs, and suggest that additional mechanisms are involved in the induction of cell death during regeneration.

### Ectopic expression of p35 in regenerating discs

**Fig 7:**
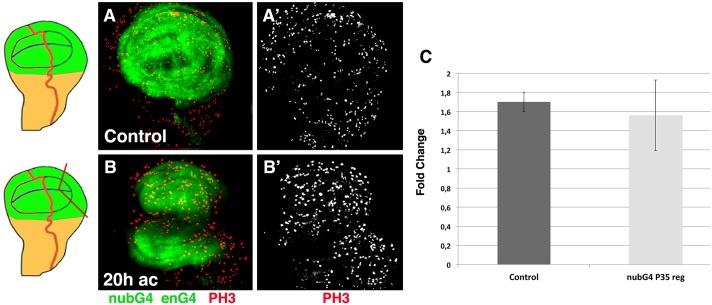
Pattern of cell proliferation in *en-Gal4 nub-Gal4 UAS-p35 UAS-GFP/+* regenerating discs. (A-B’) Third instar wing discs stained for the mitotic marker Phospho-Histone H3 (red in A-B, and grey in A’-B’). (A-A’) *en-Gal4 nub-Gal4 UAS-p35 UAS-GFP/+* control contralateral discs. (B-B’) *en-Gal4 nub-Gal4 UAS-p35 UAS-GFP/+* regenerating disc at 20 hrs AC. (C) Bar charts show the average fold change in the mitotic index of control regenerating discs (control), and *en-Gal4 nub-Gal4 UAS-p35 UAS-GFP/+* regenerating discs (nubG4 P35 Reg) at 20 hrs AC. Schematic illustrations on the left indicate the cutting lines and the regions eliminated in each disc.

Our results indicate that neither the ectopic expression of dIAP or the down-regulation of JNK was sufficient to completely eliminate apoptosis during disc regeneration. To further study the role of apoptosis during regeneration we have examined whether the over-expression of the baculoviral caspase inhibitor p35, which blocks effector caspases without affecting initiator caspases [35], is sufficient to totally block cell death during regeneration. To this end we have amputated *en-Gal4 nub*–*Gal4 UAS-p35/+* discs and analysed cell death 20 hrs AC. Previously it has been shown that in *in vivo* cultivated regenerating discs, the ectopic expression of *p35* almost completely suppressed apoptosis [18]. Consistent with this, we observed that at 20 hrs AC the number of cells expressing the apoptotic marker Caspase-3 is strongly reduced in *en-Gal4 nub*–*Gal4 UAS-p35* discs compared to control regenerating discs (S8 Fig). However, we still observed cells that express this marker in regions near to the wound as well as in regions far away from it (S8 Fig). We next examined whether the partial suppression of apoptosis observed in these discs alters the pattern of cell proliferation associated with regeneration. It has been proposed that the ectopic expression of *p35* in a context where apoptosis increases can generate undead cells. These type of cells produce mitotic signals that promote the over proliferation of surrounding tissue [36–38]. Thus, it is possible that the presence of undead cells perturb cell proliferation in these discs and the spreading of survival signals. We studied the spatial and temporal pattern of cell proliferation in *en-Gal4 nub*–*Gal4 UAS-p35/+* regenerating discs at 20 hrs AC (Fig 7). We have previously shown that at this time the density of mitotic cells in the amputated compartment was 1,7±0,1 (n = 7) higher than in non-amputated compartment. We found similar results in *en-Gal4 nub*–*Gal4 UAS-p35/+* regenerating discs, since at 20 hrs AC the mitotic density in the amputated compartment of these discs was 1,56 ±0,15 (n = 5) higher than in the non-amputated compartment (Fig 7). As previously reported for control regenerating discs, we observed that most of the mitotic cells were located in the region adjacent to the wound.

**Fig 8:**
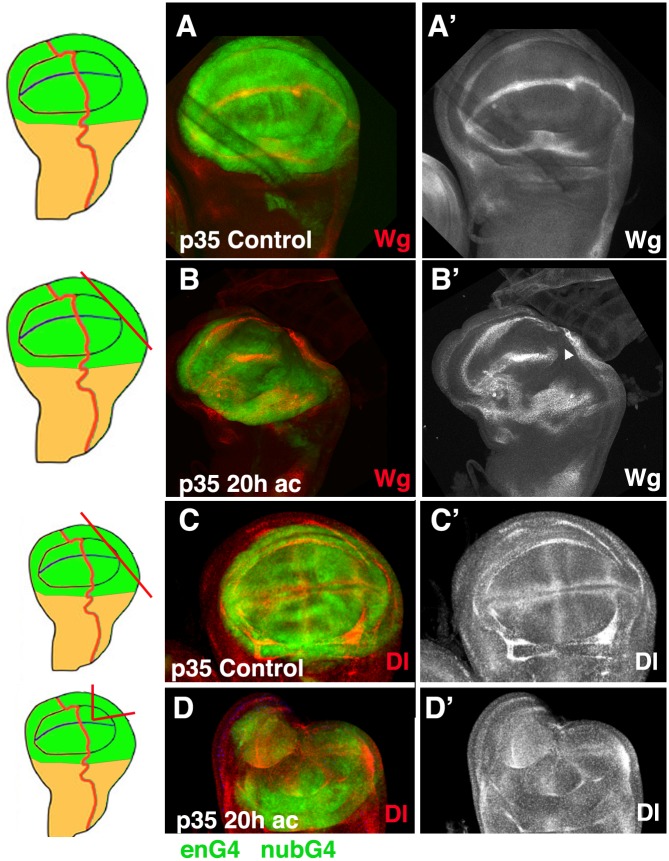
Expression of Wg and Delta in regenerating *en-Gal4 nub-Gal4 UAS-p35 UAS-GFP/+* discs 20 hrs AC. (A-B’) Expression of Wg, shown by anti-Wg (red in A-B, and grey A’-B’). (C-D’) Expression of Delta (red in C-D, and grey C’-D’). (A-A’ and C-C’) Control *en-Gal4 nub-Gal4 UAS-p35 UAS-GFP/+* third instar non amputated discs. (B-B’ and D-D’) *en-Gal4 nub-Gal4 UAS-p35 UAS-GFP/+* regenerating discs at 20hrs AC. On the left, illustrations of the discs are shown indicating the cutting lines and the regions eliminated in the discs shown by panels A-D. (B-B’) Example of an *en-Gal4 nub-Gal4 UAS-p35 UAS-GFP/+* regenerating disc at 20 hrs AC in which the expression of Wg disappears in the d/v boundary near the wound edge (arrowhead in B’). In this disc the expression of the internal ring of Wg was restored. (D-D’) The vein/intervein pattern defined by Dl was disrupted in *en-Gal4 nub-Gal4 UAS-p35 UAS-GFP/+* regenerating discs dissected 20 hrs AC (D-D’ compared to C-C’).

To further analyse whether regeneration was perturbed in *en-Gal4 nub*–*Gal4 UAS-p35/+* regenerating discs, we examined the expression of Wg. As in control regenerating discs, we observed that in 60% (n = 7) of the *en-Gal4 nub*–*Gal4 UAS-p35* regenerating discs the expression of Wg disappeared from part of the D/V boundary in the region close to the wound edge. In all these cases the down-regulation of Wg expands more than 15 cell rows from the wound site [21]) (Fig 8). As we found in control regenerating discs, in *en-Gal4 nub*–*Gal4 UAS-p35/+* regenerating discs the vein/intervein pattern is disrupted during regeneration (Fig 8) [21].

Finally, we examined the adult regenerated wings developed from *en-Gal4 nub*–*Gal4 UAS-p35/+* larvae amputated at different times during development. As observed in control regenerated wings, dependent on the time when the amputation was performed, we found different categories of regenerated adult wings. When the cut was produced late in development (140–160 Hours AEL, 0-24h before puparium formation (BPF), we find that as in controls, 100% of the adult regenerated wings displayed lack of wing tissue (n = 220 control vs n = 25 of *nub*–*Gal4 UAS-p35/+* wings) (S9 Fig). Most of the wing discs of *en-Gal4 nub*–*Gal4 UAS-p35/+* larvae amputated at earlier stages (120–140 hr AEL, 24-48h BPF), gave rise to adult wings that contain small or large nicks (86%, n = 7), and 14% of the wing analysed have completed the regeneration process and were normal compared with the control contra-lateral wing. These results are similar to those observed in control regenerated wings amputated at that stage, thus 76% of these wings contain nicks, whereas 23% have completed the regeneration process (n = 220) (in this later category we included the normal patterned regenerated wings that were smaller than control contra-lateral wing [21]).

Altogether our data indicate that the partial suppression of apoptosis produced by the ectopic expression of *p35* in regenerating discs has a minor effect in the process of regeneration.

## Discussion

Regeneration involves multiple cellular processes to restore the damaged organs or tissues. One of the mechanisms that has been proposed to play a key role as a source of signals required for regeneration in different organisms is apoptosis [11–13]. Apoptotic cells generate multiple signals that can influence the surrounding cells in different ways. The mitogenic properties of apoptotic cells are largely known; dying cells produce secreted diffusible mitogenic signals that stimulate cell proliferation [12]. In addition, recently it has been described that cell death can also induce non-autonomous cell death in neighbouring cells [20]. This process, of apoptosis-induced apoptosis can induce synchronized communal death, a mechanism that might play different roles in the extensive remodelling and morphogenetic events that take place during regeneration. Although the Drosophila imaginal discs have been used as a classical model to study regeneration, little is known about the role that apoptosis may play during disc regeneration. The main difficulty in studying the contribution of apoptosis to the regeneration of the discs relies on the different experimental approaches that have been used. The classical method to study disc regeneration—disc transplantation—causes massive cell death even in control non-amputated discs [18–19], therefore it is impossible to know whether regeneration induces cell death. The more recently developed assays of genetic ablation rely on the expression of pro-apoptotic genes in a specific region of the discs, whereby it is not possible to block cell death in the damaged region. We have used a method to study disc regeneration in physiological conditions that has allowed us to define the pattern of cell death that occurs during this process. Our results indicate that after the amputation of a fragment of the disc, cell death increases, firstly at wound edge, but as regeneration progresses apoptosis is expanded throughout the disc. Using our method, we have evaluated the contribution of cell death to the early stages of disc regeneration.

One of the limitations of our system is that the earliest time in which we can eliminate a fragment of the discs is between 96-120h AEL. We have previously shown that during this interval of time the amputated discs can fully regenerate and give rise to a completely regenerated adult wing [21]. In addition, recent results from our group suggest that at that time the different signals associated to regenerative growth such as JNK, Hippo signaling and JAK/Stat are activated (SD-G, SA and AB, in preparation). All these data suggest that in our experimental conditions, the regenerative response is similar to that described at earlier stages. However, we cannot rule out that cell death might be playing a different function during earlier stages of regeneration.

### Apoptosis-induced apoptosis mechanism during disc regeneration

The pattern of cell death that we have observed in regenerating discs is consistent with a model in which dead cells generated at the wound edge can induce cell death throughout the entire disc by an apoptosis-induced apoptosis mechanism. It has been shown that this process relies on the production of Eiger by the dying cells, which activates the JNK pathway in neighbouring cells, inducing them to die [20]. The evolution of the activity of this signaling pathway during disc regeneration, firstly at the wound edge and later in most of the cells of the wing pouch, is consistent with this model [19] (S4 and S5 Figs). However, our observations suggest that although an apoptosis-induced apoptosis mechanism mediated by *eiger* might be involved in promoting cell death in regenerating discs, other signals and mechanisms are also required. Thus, we have found that cell death is not completely abolished in regenerating *eiger* mutant discs, as occurs after cell death induction [20]. In addition, the ectopic activation of *eiger* is not sufficient to induce the high number of “non autonomous”dead cells found in regenerating discs. These data suggest that during regeneration, either there are apoptotic signals that are independent of JNK signalling, or there are additional mechanisms, independent of *eiger*, that trigger JNK signaling. Interestingly, we found that in *eiger* mutant discs, JNK signaling is active, though mostly in the cells around the wound edge. This result implies that during regeneration, the JNK pathway is activated by an alternative way to Eiger, at least in some cells. Previous studies have demonstrated that loss of epithelial integrity induces JNK signaling. This might be mediated by proteins like the small GTPase, Rho1, that is known to control epithelial morphogenesis and integrity through its ability to regulate the cytoskeleton, and can promote JNK pathway activity through an interaction with Slpr, an upstream component of the JNK pathway [32]. Thus, the epithelial damage caused by the amputation could initially activate JNK signalling through a mechanism independent of *eiger*, and therefore induce cell death, even in absence of *eiger*. However, the fact that the activity of JNK signalling in *eiger* mutant regenerating discs is mostly restricted to the wound edges, though we found apoptotic cells far away from this site, and that the ectopic activation of JNK signalling is not sufficient to reproduce the strong increase “non-autonomous” cell death observed during disc regeneration, suggests that apoptotic signals independent of JNK signaling are triggered during regeneration. Recently, it has been reported that after inducing cell death or as consequence of the mechanical stress generated during wounding, imaginal disc cells produce reactive oxygen species (ROS) [39]. ROS, either by diffusing from cell to cell or by propagating their production in surrounding cells, function as a paracrine signal that can stimulate the activity of stress-activated protein kinases such as, p38 and JNK stress MAP kinases [39]. Thus, ROS production might result in extensive spreading of apoptotic signals to adjacent cells through activation of these signalling pathways, and likely through other undefined death pathways that may trigger apoptosis independent of JNK signalling. In this scenario, the elimination of *eiger* would not be sufficient to block all the apoptosis during discs regeneration.

### Apoptosis-induced proliferation mechanism during disc regeneration

This process seems best suited to aid complete regeneration, since dying cells generate after amputation might promote cell proliferation of the surrounding tissue to restore the tissue lost after damage. The nature of these signaling molecules differs depending on the tissue [12]. Different reports using distinct experimental approaches to study disc regeneration have led to divergent conclusions about the mitogenic signals involved in regeneration [8–7,40–41]. Thus, whereas some authors have proposed that the ortholog of mammalian Wnt, Wingless (Wg) is necessary for imaginal disc regeneration, other authors have showed that Wg is not required for this process [7,21,40–41]. Therefore, the role of apoptosis-induced proliferation and the molecules involved in this process as a compensatory mechanism during disc regeneration remains unresolved. Here we have shown that a partial suppression of cell death during regeneration by over-expressing p35 does not have any measurable effect on cell proliferation during early stages of regeneration. We have also partially inhibited cell death in regenerating discs by down-regulating JNK signalling. Previously, it has been shown that mutations of members of this pathway strongly reduce the number of mitotic cells in regenerating discs [18–19]. Considering the multiple functions of JNK signaling during regeneration [19,27–30], and that the down-regulation of this pathway disrupts wound healing and regeneration in wing discs (see results) [19], the effect on cell proliferation could be indirect and not a consequence of the reduced apoptosis.

In summary our results suggest that apoptosis does not seem to play an important role inducing cell proliferation during regeneration, since partial suppression of cell death does not affect cell proliferation at early stages of regeneration. However, as we have not completely inhibited cell death in regenerating discs, we cannot rule out that programmed cell death contributes to effectively complete disc regeneration.

The variety of processes that take place during regeneration, such as remodelling, migration and, reorganization of epithelia, likely requires the elimination of “unwanted cells”. This could be achieved by the action of different cell death-induced mechanisms that might co-operate during regeneration, increasing the robustness of the process. Then it is possible that the mechanical stress and tissue tensions generated during amputation, in addition to the activation of signaling pathways previously mentioned, also might trigger new unidentified signals to promote apoptosis and/or other mechanisms of programmed cell death, such as autophagy and necroptosis. Functional redundancy of some of these mechanisms could explain the difficulty in completely eliminating cell death during regeneration. This would be consistent with our observations which indicate that the inhibition of only one pro-apoptotic pathway is not sufficient to block cell death during regeneration.

## Supporting Information

S1 Fig Pattern of cell death during wing disc regeneration. (A-D’) Third instar wing *en-Gal4 UAS-GFP* discs stained for the apoptotic marker anti-cleaved Caspase-3 (red in A-D, and grey in A’-D’). (A-A’) Control discs. (B-B’) Regenerating discs at 3 hrs after cut (AC). We only observed dead cells in the wound edge or in the region adjacent. (C-C’) Regenerating discs at 6 hrs AC; we observed a significant increase in the number of dead cells in the posterior as well as the anterior compartments. (D-D’) 20 hrs AC, we observed a high number of apoptotic cells in the region near to the wound edge, as well as in the anterior compartment. Note the cluster of dead cells in the anterior compartment. Schematic illustrations on the left indicate the cutting lines and the regions eliminated in each disc.
(TIF)

S2 Fig Pattern of cell death during wing disc regeneration. (A-F”) Third instar wing *en-Gal4 UAS-GFP* regenerating discs double staining for the apoptotic marker anti-cleaved Caspase-3 (Blue in A,C, E and F) and DAPI (red in A,C,E and F) at 20hrs AC. (A-B) Regenerating discs at 20 hrs AC; we observed dead cells in the posterior as well as in the anterior compartments. (C-D) Higher magnification of the panels (A-B), note the cluster of dead cells in the anterior compartment. These apoptotic anterior cells, to difference to posterior apoptotic cells, do not express GFP (Arrows). (E-F”) Y-Z projections show a cross-section at the position of the white line in the posterior compartment (E-E”) or the anterior compartment green line (F-F”). We observe Caspase-3 positive cells in the anterior compartment that are integrated in the columnar epithelium (arrowhead in F). Posterior apoptotic cells express GFP (arrow in E).
(TIF)

S3 Fig Pattern of proliferation in *en-Gal4 UAS-GFP nub-Gal4; UAS-DIAP1/+* regenerating discs. (A-B”) Third instar wing discs stained for the mitotic marker Phospho-Histone H3 (blue in A-B, and grey in A”-B”) and anti-Wg (red in A-B, and grey in A’-B’). (A-A”) *en-Gal4 UAS-GFP nub-Gal4; UAS-DIAP1/+* control non-amputated contra-lateral discs. (B-B”) *en-Gal4 UAS-GFP nub-Gal4; UAS-DIAP1/+* regenerating disc at 20 hrs AC. Cell proliferation increases in the posterior compartment of these discs. (C) Bar charts show the average fold change in the mitotic index of control regenerating discs (control), and *en-Gal4 UAS-GFP nub-Gal4; UAS-DIAP1/+* regenerating discs (*nubG4 dIAP1* reg) at 20 hrs AC, compared to control non-regenerating discs. The error bars represent the standard deviation. Schematic illustrations on the left indicate the cutting lines and the regions eliminated in each disc.
(TIF)

S4 Fig Expression of *puc-LacZ* reporter in *eiger*^*3*^*/eiger*^*1*^ regenerating discs at 6 hrs AC. (A- A”) Third instar *eiger*^*3*^*/eiger*^*1*^; *pucLacZ/+* control non-amputated discs. (B-B”) Third instar *pucLacZ/+* amputated discs. (C-C”) *eiger*^*3*^*/eiger*^*1*^; *puc-LacZ /+* amputated discs. The discs were cultivated during 6 hrs after amputation (see M&M). The discs were stained with phalloidin (red in A–C, and grey A’-C’); and anti-ß-Galactosidase (green in A-C and grey in A”-C”) to reveal the pattern of expression of JNK reporter *puc-lacZ*. In *eiger*^*3*^*/eiger*^*1*^mutant discs the expression of the reporter is mostly restricted to the wound edges (compared C-C” with B-B”). Optical z-sections below the panels showed a cross-section at the position of the white line. Note that to difference to control discs, that have completed the wound healing process (arrows in Optical z-section in B-B’) and the epithelial integrity is restored, in *eiger*^*3*^*/eiger*^*1*^mutants the wound is unhealed, and the epithelial is disrupted at the wound site (arrowheads in Optical z-sections C-C”).
(TIF)

S5 Fig Expression of *puc-LacZ* reporter in *eiger*^*3*^*/eiger*^*1*^ regenerating discs at 20 hrs AC. (A- A’) Third instar *Hh-dsRed Ci-Gal4 UAS-GFP*/ *puc-LacZ* non-amputated control discs. (B-B’) Third instar *en-Gal4 UAS-GFP*/ puc-LacZ amputated discs. (C-C’) *eiger*^*3*^*/eiger*^*1*^; *Hh-dsRed Ci-Gal4 UAS-GFP/puc-LacZ /+* amputated discs. The discs were analysed 20 hrs AC. The discs were stained with anti-ß-Galactosidase (red in A-C and grey in A’-C’) to reveal the pattern of expression of JNK reporter *puc-lacZ*. In *eiger*^*3*^*/eiger*^*1*^mutant discs the expression of the reporter is mostly restricted to the wound edges (compared C with B).
(TIF)

S6 Fig Apoptosis is reduced in *egr*^*1*^*/egr*^*3*^ regenerating discs. (A-A”) Third instar *eiger*^*3*^*/eiger*^*1*^; *Hh-dsRed Ci-Gal4 UAS-GFP/+* discs wing stained for the apoptotic marker anti-cleaved Caspase-3 (blue in A and A”, and grey in A’) and anti-Wg (red in A and A”), at 20 hrs AC.
(TIF)

S7 Fig Expression of Wg in regenerating *en-Gal4 UAS-puc UAS-GFP/+* discs 20 hrs AC. (A-C”) Expression of Wg, stained with anti-Wg (red in A-C, and grey A’-C”) in control third instar non amputated discs (A-A”), control amputated discs (B-B”) and *en-Gal4 UAS-puc UAS-GFP/+* regenerating discs at 20 hrs AC (C-C”). (C-C”) In *en-Gal4 UAS-puc UAS-GFP/+* regenerating discs 20 hrs AC the expression of Wg does not disappears at the d/v boundary as it occurs in control regenerating discs (B-B”).
(TIF)

S8 Fig Apoptotic pattern in *en-Gal4 nub-Gal4 UAS-p35 UAS-GFP/+* regenerating discs. (A-B’) Discs stained for the apoptotic marker anti-cleaved Caspase-3 (red in A-B, and grey in A’-B’). (A-A’) Control *en-Gal4 nub-Gal4 UAS-p35 UAS-GFP/+* non-amputated discs. (B-B’) *en-Gal4 nub-Gal4 UAS-p35 UAS-GFP/+* regenerating discs at 20 hrs AC. We observed that cell dead in reduced compared to control regenerating discs (compared to Fig 1).
(TIF)

S9 Fig Regenerated adult *en-Gal4 nub-Gal4 UAS-p35 UAS-GFP/+* wings. (A) Different examples of adult regenerated *en-Gal4 nub-Gal4 UAS-p35 UAS-GFP/+* wings, and control contralateral wings (lower wings, Wt). The discs were amputated at different times during development. Depending on the size of the fragment amputated and time passed BPF (left) we observed a range of regenerated adult wing phenotypes.
(TIF)
