# Supplementary figures and images for: Analysis of the Function of Apoptosis during Imaginal Wing Disc Regeneration in Drosophila melanogaster
Source: PLoS One. 2016 Nov 28;11(11):e0165554. doi: 10.1371/journal.pone.0165554 (PMC5125585; doi:10.1371/journal.pone.0165554)

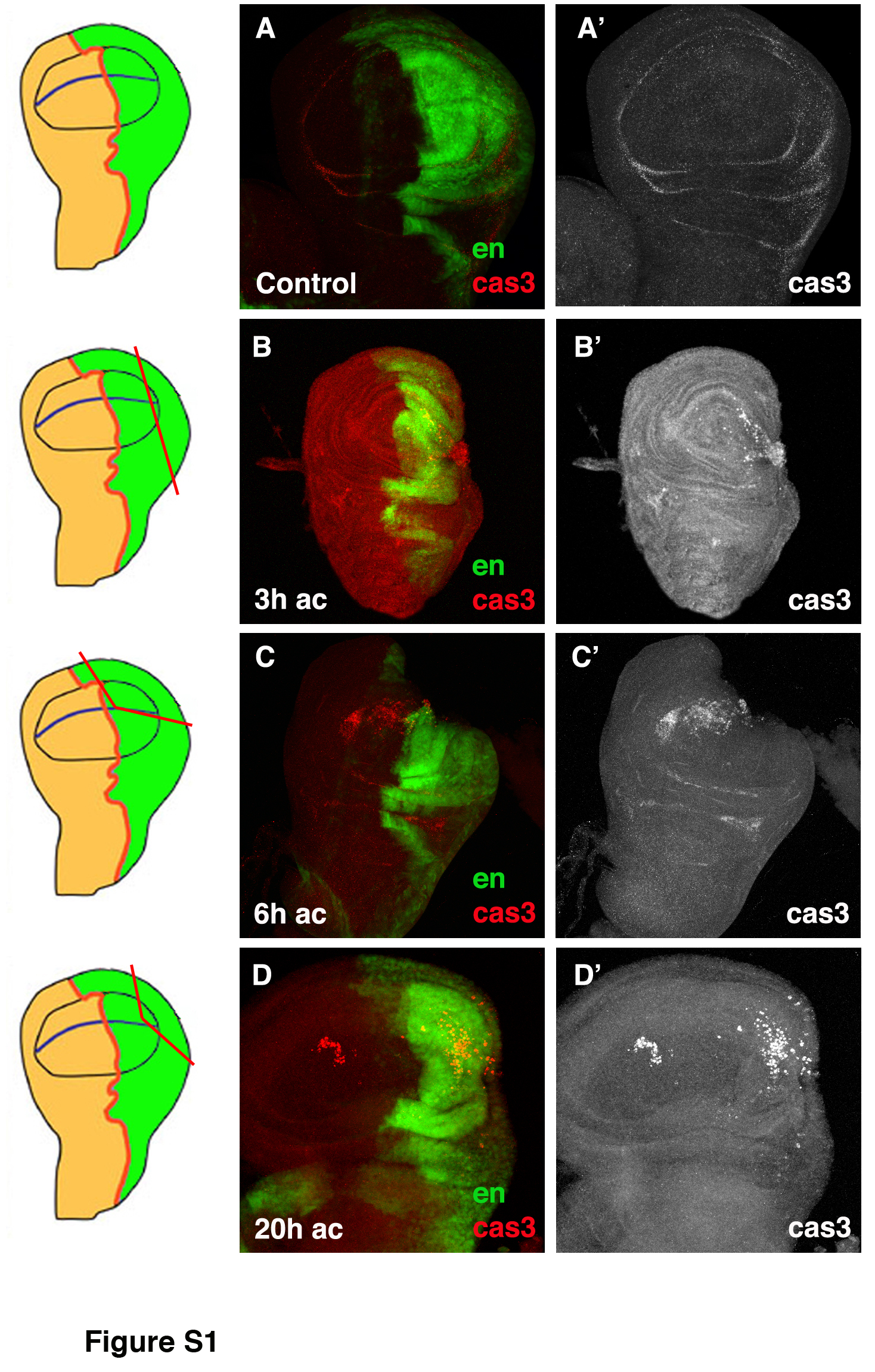

Supplement: S1 Fig — (A-D’) Third instar wing en-Gal4 UAS-GFP discs stained for the apoptotic marker anti-cleaved Caspase-3 (red in A-D, and grey in A’-D’). (A-A’) Control discs. (B-B’) Regenerating discs at 3 hrs after cut (AC). We only observed dead cells in the wound edge or in the region adjacent. (C-C’) Regenerating discs at 6 hrs AC; we observed a significant increase in the number of dead cells in the posterior as well as the anterior compartments. (D-D’) 20 hrs AC, we observed a high number of apoptotic cells in the region near to the wound edge, as well as in the anterior compartment. Note the cluster of dead cells in the anterior compartment. Schematic illustrations on the left indicate the cutting lines and the regions eliminated in each disc. (TIF) [file pone.0165554.s001.tif]

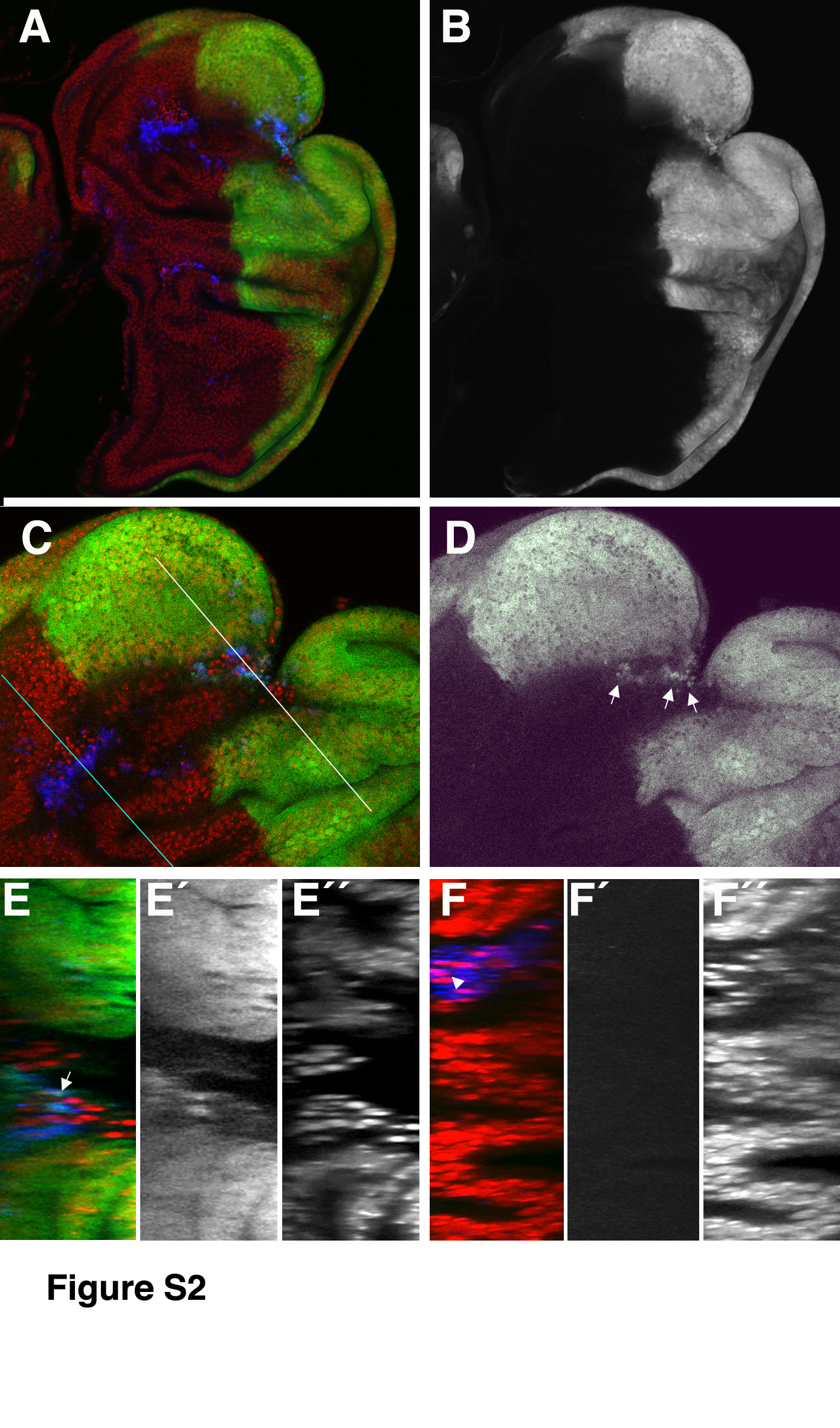

Supplement: S2 Fig — (A-F”) Third instar wing en-Gal4 UAS-GFP regenerating discs double staining for the apoptotic marker anti-cleaved Caspase-3 (Blue in A,C, E and F) and DAPI (red in A,C,E and F) at 20hrs AC. (A-B) Regenerating discs at 20 hrs AC; we observed dead cells in the posterior as well as in the anterior compartments. (C-D) Higher magnification of the panels (A-B), note the cluster of dead cells in the anterior compartment. These apoptotic anterior cells, to difference to posterior apoptotic cells, do not express GFP (Arrows). (E-F”) Y-Z projections show a cross-section at the position of the white line in the posterior compartment (E-E”) or the anterior compartment green line (F-F”). We observe Caspase-3 positive cells in the anterior compartment that are integrated in the columnar epithelium (arrowhead in F). Posterior apoptotic cells express GFP (arrow in E). (TIF) [file pone.0165554.s002.tif]

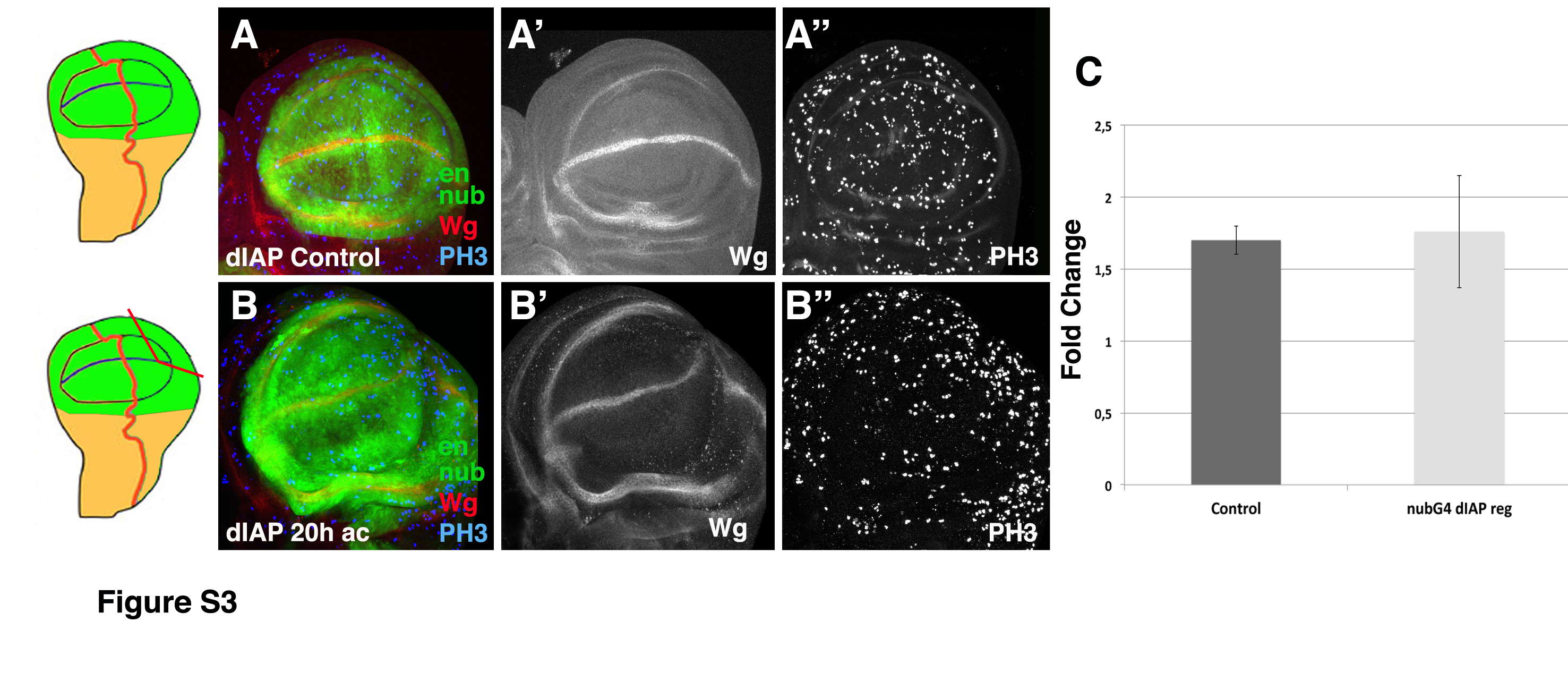

Supplement: S3 Fig — (A-B”) Third instar wing discs stained for the mitotic marker Phospho-Histone H3 (blue in A-B, and grey in A”-B”) and anti-Wg (red in A-B, and grey in A’-B’). (A-A”) en-Gal4 UAS-GFP nub-Gal4; UAS-DIAP1/+ control non-amputated contra-lateral discs. (B-B”) en-Gal4 UAS-GFP nub-Gal4; UAS-DIAP1/+ regenerating disc at 20 hrs AC. Cell proliferation increases in the posterior compartment of these discs. (C) Bar charts show the average fold change in the mitotic index of control regenerating discs (control), and en-Gal4 UAS-GFP nub-Gal4; UAS-DIAP1/+ regenerating discs (nubG4 dIAP1 reg) at 20 hrs AC, compared to control non-regenerating discs. The error bars represent the standard deviation. Schematic illustrations on the left indicate the cutting lines and the regions eliminated in each disc. (TIF) [file pone.0165554.s003.tif]

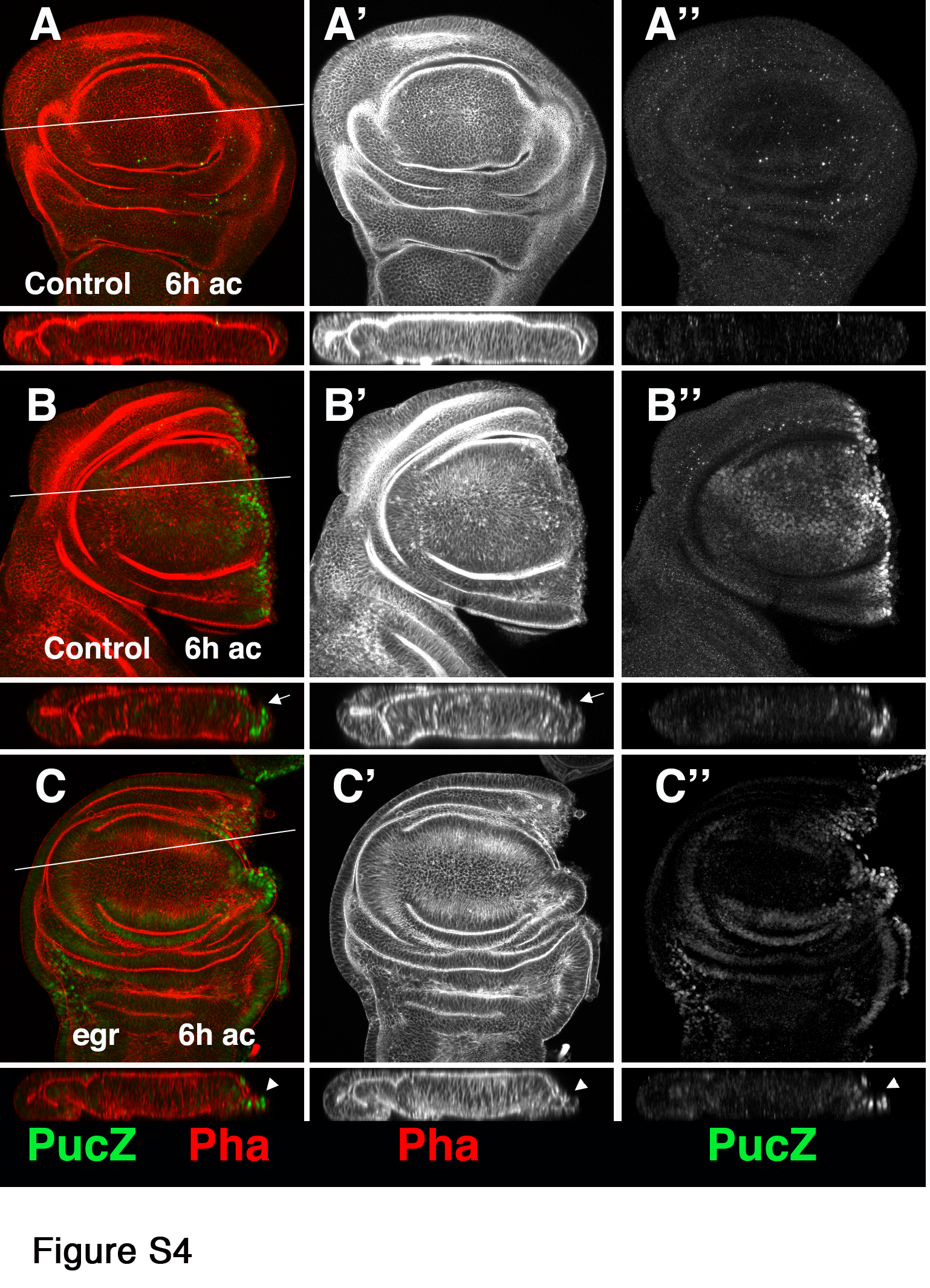

Supplement: S4 Fig — (A- A”) Third instar eiger3/eiger1; pucLacZ/+ control non-amputated discs. (B-B”) Third instar pucLacZ/+ amputated discs. (C-C”) eiger3/eiger1; puc-LacZ /+ amputated discs. The discs were cultivated during 6 hrs after amputation (see M&M). The discs were stained with phalloidin (red in A–C, and grey A’-C’); and anti-ß-Galactosidase (green in A-C and grey in A”-C”) to reveal the pattern of expression of JNK reporter puc-lacZ. In eiger3/eiger1mutant discs the expression of the reporter is mostly restricted to the wound edges (compared C-C” with B-B”). Optical z-sections below the panels showed a cross-section at the position of the white line. Note that to difference to control discs, that have completed the wound healing process (arrows in Optical z-section in B-B’) and the epithelial integrity is restored, in eiger3/eiger1mutants the wound is unhealed, and the epithelial is disrupted at the wound site (arrowheads in Optical z-sections C-C”). (TIF) [file pone.0165554.s004.tif]

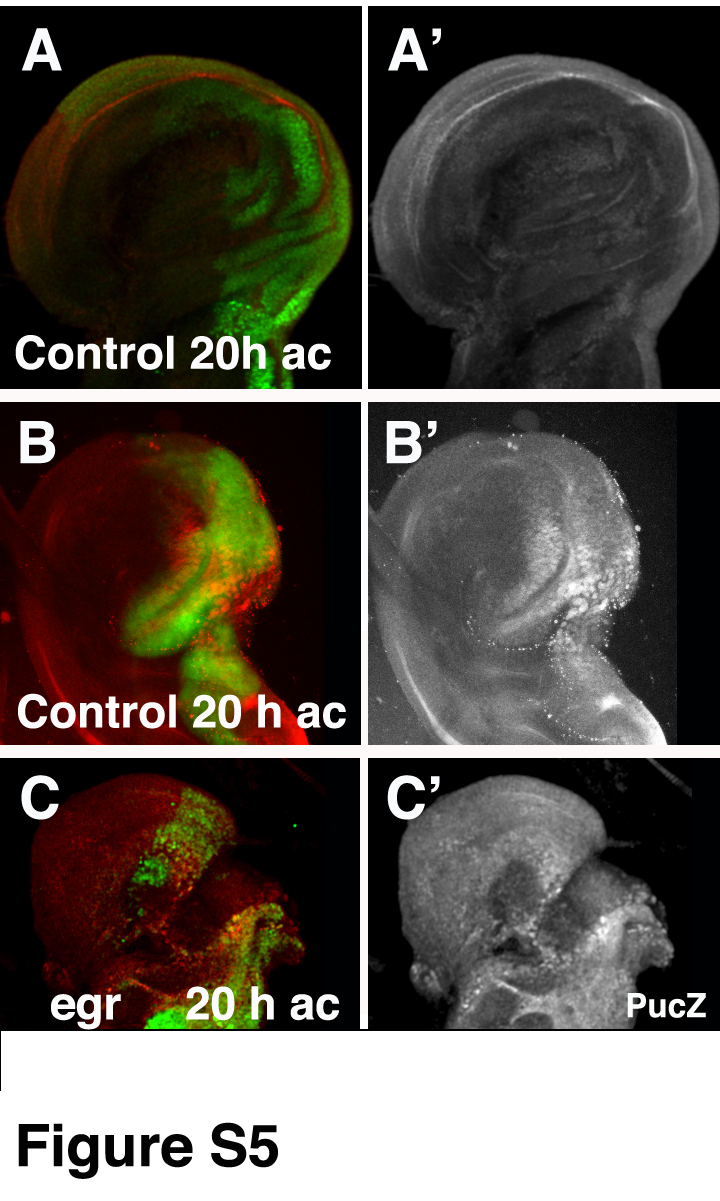

Supplement: S5 Fig — (A- A’) Third instar Hh-dsRed Ci-Gal4 UAS-GFP/ puc-LacZ non-amputated control discs. (B-B’) Third instar en-Gal4 UAS-GFP/ puc-LacZ amputated discs. (C-C’) eiger3/eiger1; Hh-dsRed Ci-Gal4 UAS-GFP/puc-LacZ /+ amputated discs. The discs were analysed 20 hrs AC. The discs were stained with anti-ß-Galactosidase (red in A-C and grey in A’-C’) to reveal the pattern of expression of JNK reporter puc-lacZ. In eiger3/eiger1mutant discs the expression of the reporter is mostly restricted to the wound edges (compared C with B). (TIF) [file pone.0165554.s005.tif]

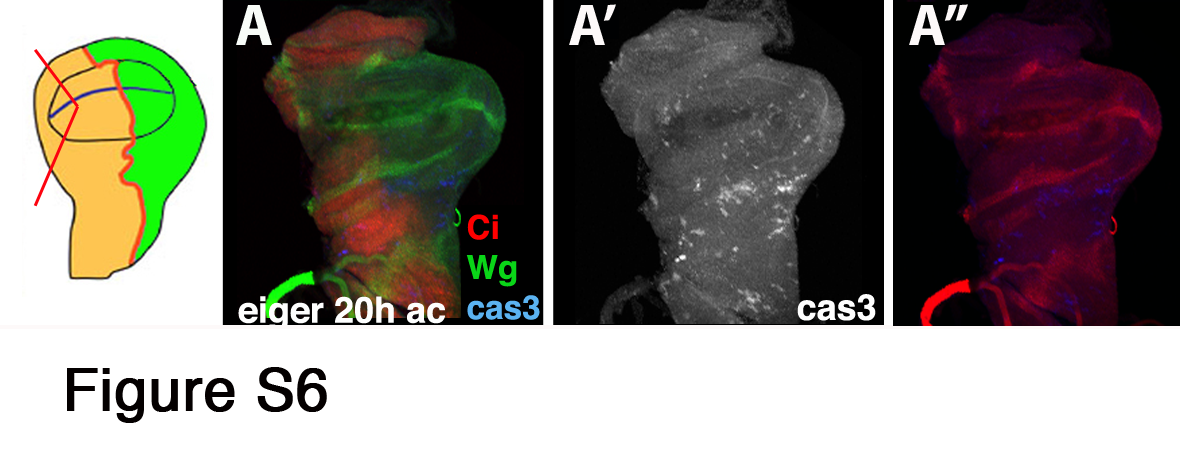

Supplement: S6 Fig — (A-A”) Third instar eiger3/eiger1; Hh-dsRed Ci-Gal4 UAS-GFP/+ discs wing stained for the apoptotic marker anti-cleaved Caspase-3 (blue in A and A”, and grey in A’) and anti-Wg (red in A and A”), at 20 hrs AC. (TIF) [file pone.0165554.s006.tif]

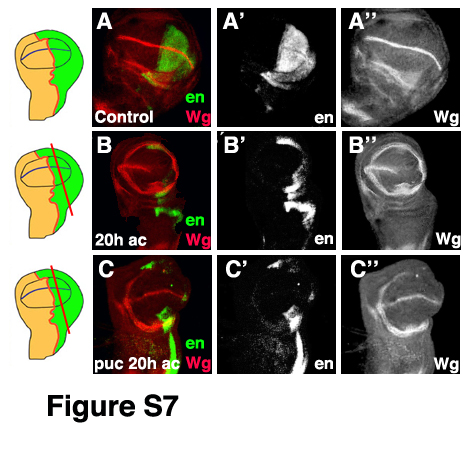

Supplement: S7 Fig — (A-C”) Expression of Wg, stained with anti-Wg (red in A-C, and grey A’-C”) in control third instar non amputated discs (A-A”), control amputated discs (B-B”) and en-Gal4 UAS-puc UAS-GFP/+ regenerating discs at 20 hrs AC (C-C”). (C-C”) In en-Gal4 UAS-puc UAS-GFP/+ regenerating discs 20 hrs AC the expression of Wg does not disappears at the d/v boundary as it occurs in control regenerating discs (B-B”). (TIF) [file pone.0165554.s007.tif]

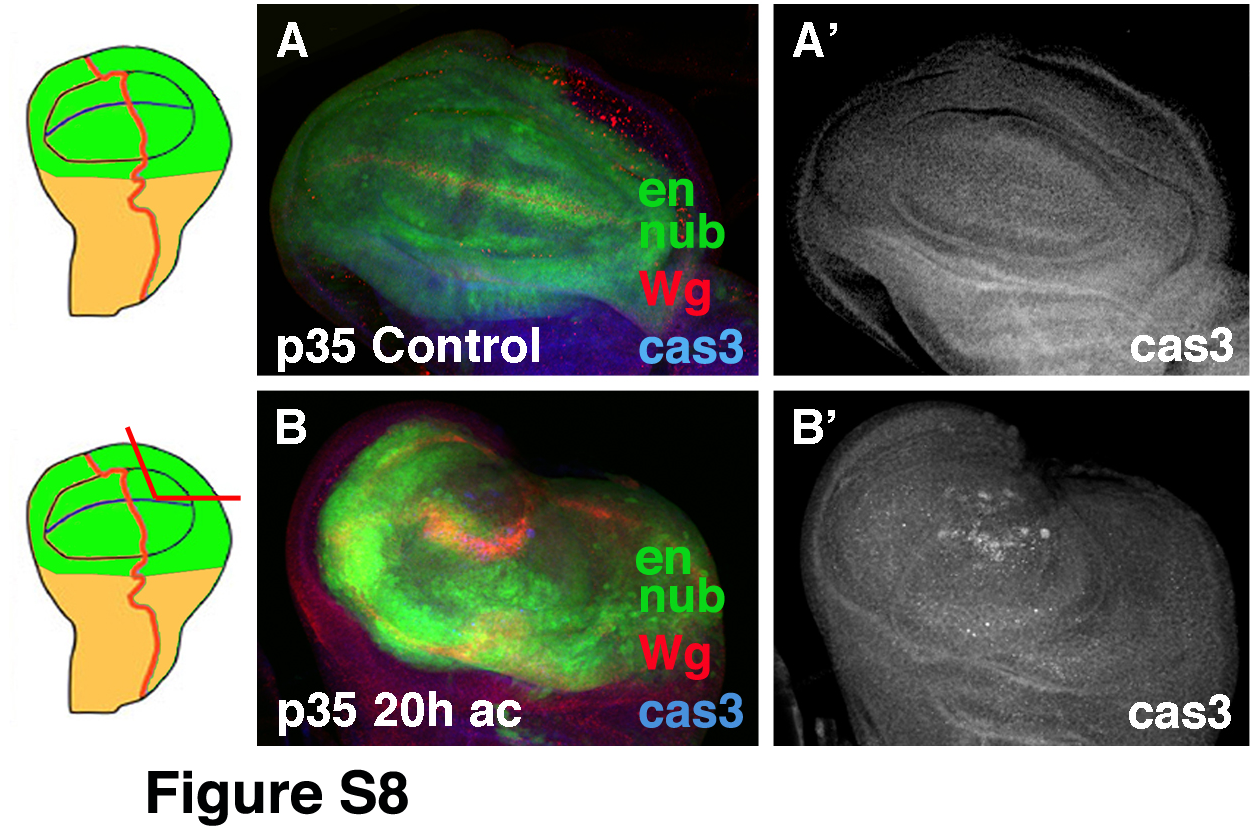

Supplement: S8 Fig — (A-B’) Discs stained for the apoptotic marker anti-cleaved Caspase-3 (red in A-B, and grey in A’-B’). (A-A’) Control en-Gal4 nub-Gal4 UAS-p35 UAS-GFP/+ non-amputated discs. (B-B’) en-Gal4 nub-Gal4 UAS-p35 UAS-GFP/+ regenerating discs at 20 hrs AC. We observed that cell dead in reduced compared to control regenerating discs (compared to Fig 1). (TIF) [file pone.0165554.s008.tif]

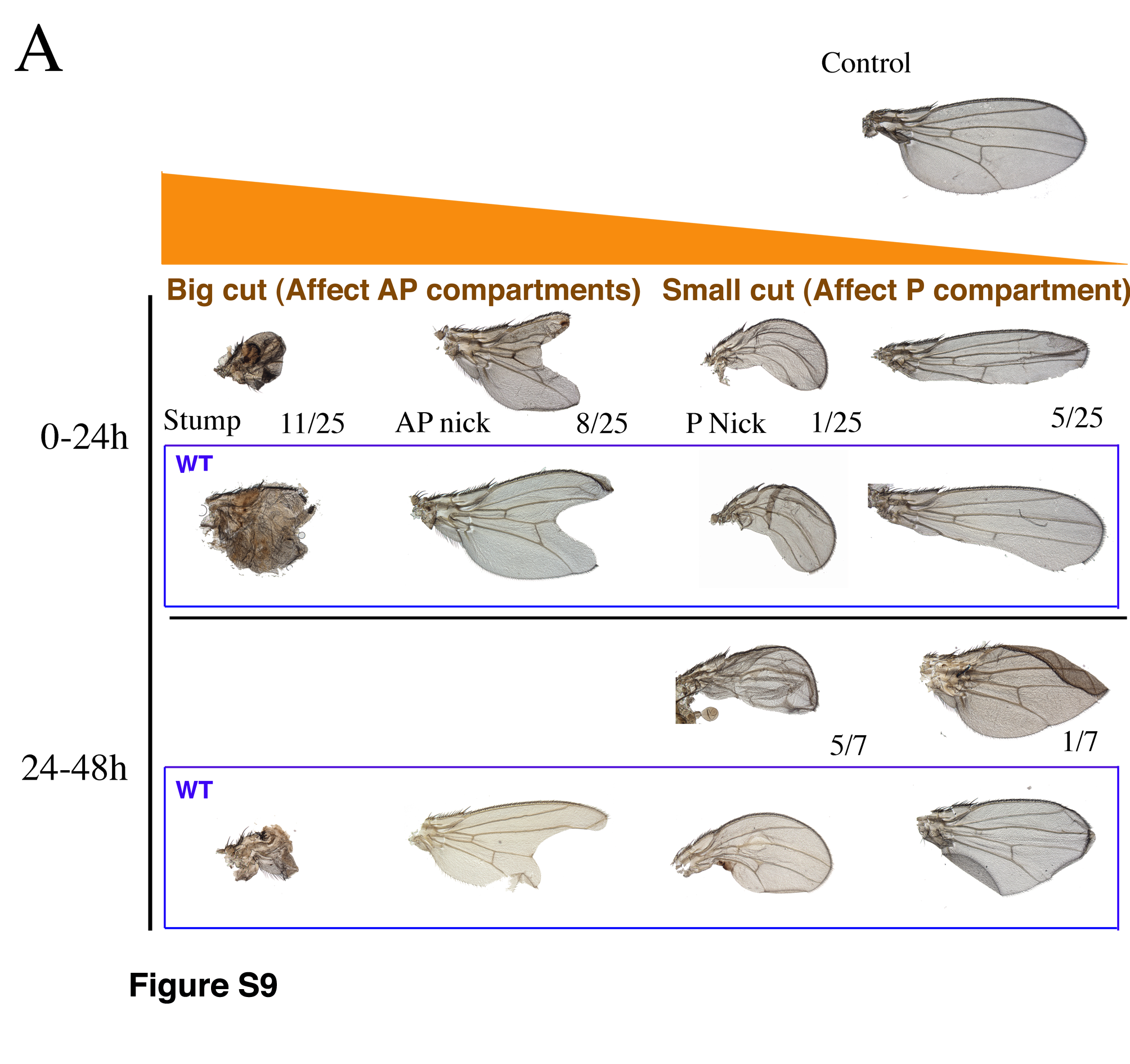

Supplement: S9 Fig — (A) Different examples of adult regenerated en-Gal4 nub-Gal4 UAS-p35 UAS-GFP/+ wings, and control contralateral wings (lower wings, Wt). The discs were amputated at different times during development. Depending on the size of the fragment amputated and time passed BPF (left) we observed a range of regenerated adult wing phenotypes. (TIF) [file pone.0165554.s009.tif]
